# Retroperitoneal Peripancreatic Ganglioneuroma Encasing the Celiac Trunk and Superior Mesenteric Artery

**DOI:** 10.7759/cureus.52405

**Published:** 2024-01-16

**Authors:** Pablo Avila-Sanchez, Natalia M Barron-Cervantes, Alejandro Martinez-Esteban, Luis C Chan-Nuñez

**Affiliations:** 1 Department of Hepato-Pancreato-Biliary Surgery, Instituto Nacional de Ciencias Medicas y Nutrición Salvador Zubiran, Mexico City, MEX; 2 Department of General and Gastrointestinal Surgery, Medica Sur Hospital, Mexico City, MEX; 3 Department of General and Gastrointestinal Surgery, Fundación Clínica Medica Sur, Mexico City, MEX; 4 Department of Hepato-Pancreato-Biliary Surgery, Instituto Nacional de Ciencias Medicas y Nutricion Salvador Zubiran, Mexico City, MEX

**Keywords:** superior mesenteric artery, celiac trunk, encasement, ganglioneuroma, peripancreatic, retroperitoneal

## Abstract

A retroperitoneal ganglioneuroma is an exceptionally rare surgical entity, even more so in pancreaticoduodenal tumors. These well-differentiated neuroepithelial tumors originate in the neural crest, emerge in the sympathetic nervous system, and consist of ganglion cells and stromal Schwann cells. Generally, these tumors, despite being mostly benign, may be associated with venous or arterial vascular involvement. The symptomatology presented will depend on the mass effect due to tumor growth, and surgical excision is the only therapeutic option offered today to these patients. However, encapsulation of the main vessels represents a great surgical complexity. Various surgical approaches have been employed throughout history; however, the current preferred method is an open midline laparotomy, involving an extensive Kocher maneuver and an artery-first approach, aiming for an R0 resection of the tumor with total vascular preservation to the greatest extent possible. We present a case of an R2 resection involving a 95 mm x 85 mm retroperitoneal peripancreatic ganglioneuroma with double vascular involvement (celiac trunk and superior mesenteric artery). The procedure utilized an artery-first approach with total vascular preservation in a 17-year-old woman who had long-standing gastrointestinal symptoms due to the mass effect.

## Introduction

Ganglioneuromas are rare benign, well-differentiated neuroepithelial tumors originating from the neural crest. They arise in the sympathetic nervous system and are composed of ganglion cells and stromal Schwann cells. They can appear in any location, from the base of the skull to the pelvis, with the adrenal glands being the most frequent location [1]. The incidence reported of ganglioneuromas is one per 1,000,000 people. However, as a primary retroperitoneal tumor, it only constitutes a small percentage of all cases of primary retroperitoneal tumors (0.7%-1.6%) [2]. Its preoperative diagnosis is often missed as it is typically based on histopathological findings. Nevertheless, a simple or contrasted computerized tomography (CT) scan is the most useful imaging modality, revealing the extent of the tumor, organ of origin, regional invasion, vascular encasement, or adenopathies if present. Also, it is important to mention that transoperative biopsies allow for a more complete surgical approach. Surgical removal is the only option offered today to these patients; however, encasement of major vessels represents a great surgical complexity. Many surgical approaches have been used throughout medical history, but currently, the preferred method involves an artery-first approach to tumor resection, aiming for an R0 resection and leaving no tumor remnant [3]. Our case details a patient with a retroperitoneal peripancreatic ganglioneuroma involving the encasement of the celiac trunk and superior mesenteric artery. The treatment occurred at a first-level hepatopancreatic-biliary surgical center in Mexico City. This case is presented to further expand knowledge about the possible clinical presentation of this condition, enabling early diagnosis. This information empowers other surgeons to consider this tumor, helping prevent fatal outcomes.

## Case presentation

**Figure 1:**
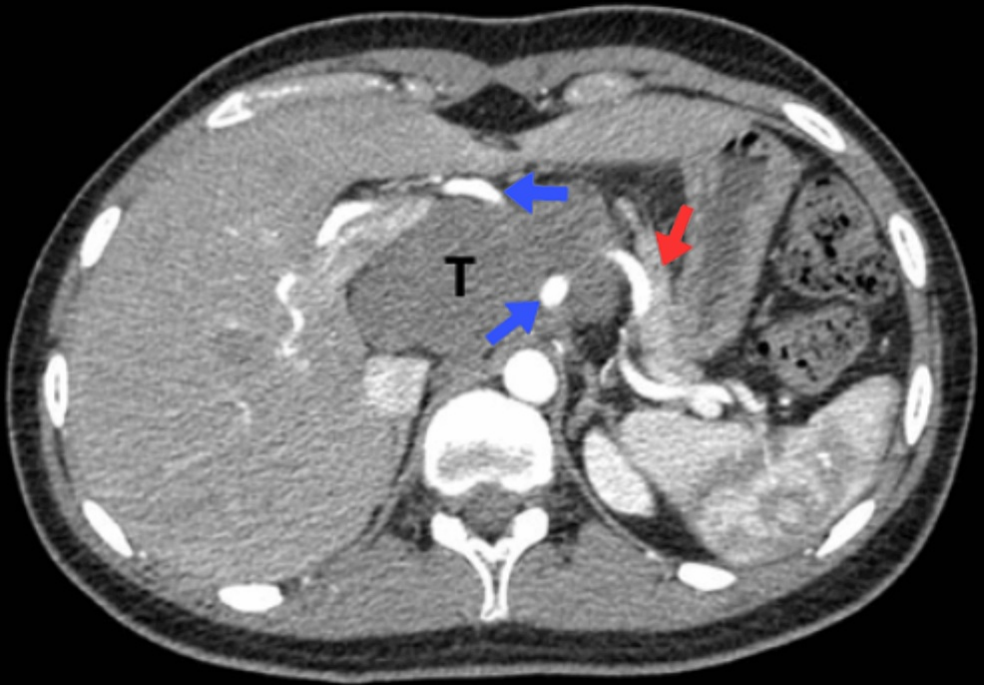
Contrasted abdominal computed axial tomography in the arterial phase. A retroperitoneal tumor (T) is observed without contrast enhancement, with displacement of the body and tail of the pancreas (red arrow), which surrounds the celiac trunk and its branches (blue arrows).

A 17-year-old female presented to the emergency room with the chief complaint of postprandial fullness and nonspecific abdominal distension for the last five months. In her relevant past medical history, she received a diagnosis of polycystic ovarian syndrome. During the physical examination, she exhibited abdominal distension that was non-painful upon palpation. There were no alterations in peristaltic sounds, and no signs suggestive of peritoneal irritation were observed. Because of the chief complaint an abdominal ultrasound (US) was performed with a report of increased volume of the pancreas at the expense of a hypoechoic image. During her time in the emergency room, an intravenous contrast CT was performed. The report indicated a hypodense mass measuring 95 mm x 85 mm with an irregular shape and well-defined margins, originating from the head of the pancreas. The mass showed no significant enhancement and caused displacement of the body and tail of the pancreas. It surrounded the vascular structures of the celiac trunk without compromising its caliber (Figure 1). Following the radiological findings, an endoscopic EUS was conducted. The report indicated a solid tumor originating from the head and neck of the pancreas, displaying hypoechoic and heterogeneous characteristics. The tumor caused displacement of the splenic artery and superior mesenteric artery. During the procedure, a guided biopsy of the tumor was performed. However, the endoscopic US biopsies were negative for any pathological process, so a US-guided percutaneous biopsy was performed with a histological report of ganglioneuroma.

**Figure 2:**
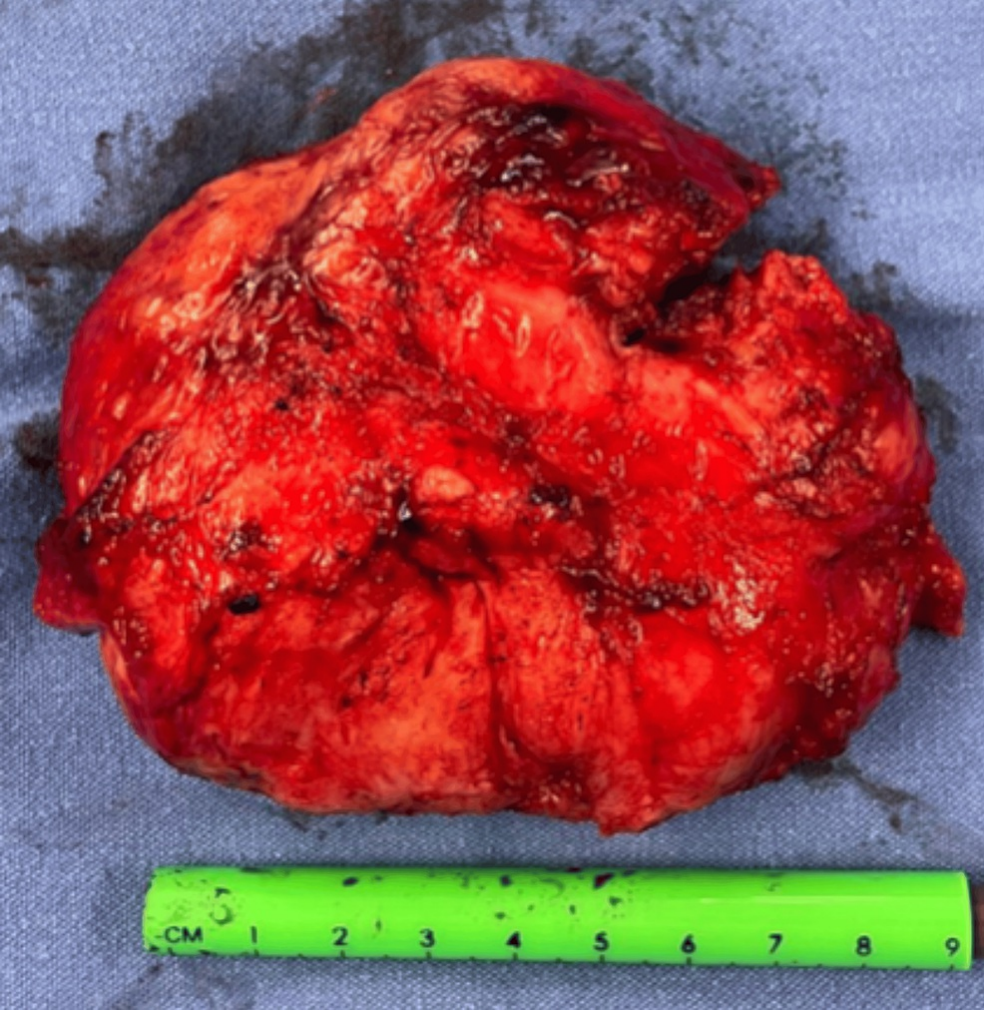
Surgical piece. Retroperitoneal ganglioneuroma measuring 9 cm x 8 cm.

Surgical treatment was initially planned as an open pancreaticoduodenectomy with Billroth II reconstruction. However, during the surgical procedure and after duodenal mobilization, it was confirmed that the mass did not entirely originate from the pancreas. Consequently, the decision was made to opt for a complete resection of the lesion with vascular dissection and preservation of the pancreas and duodenum (Figure 2).

**Figure 3:**
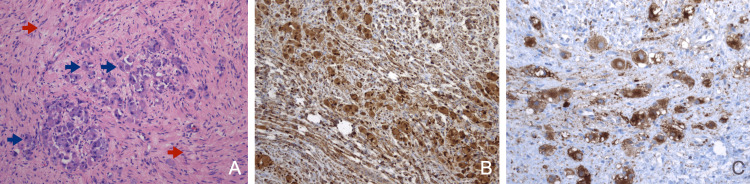
Histopathological features. (A) The Schwannian stroma is composed of uniform, spindle cells grouped in fascicles (red arrows); the mature ganglion cells (blue arrows) correspond to abundant eosinophilic cytoplasm and an eccentric basophilic nucleus. (B) S100 stains both ganglion cells and Schwannian stroma. (C) Ganglion cells are positive for synaptophysin.

During the surgical resection, tumor encasement of the superior mesenteric artery was observed, which was dissected adequately using an *artery-first approach* without complications. Additionally, encasement of the celiac trunk and its principal branches was discovered, posing challenges for resection at the emergence of the common hepatic artery. It was decided to ligate it once adequate hepatic hematic reflux through the gastroduodenal artery was confirmed. An R2 resection was performed at the level of the celiac axis, leaving a tumor remnant at the level of the pancreatic head. Finally, one drainage was left in the abdominal cavity. The histopathology report showed that the tumor contained Schwannian stroma (>50%) with mature ganglion cells, and no maturing neuroblasts/gangliocytes were identified. Both components, the Schwannoid stroma and the ganglion cells, were diffusely positive in immunohistochemistry for protein S100. Additionally, only the ganglion cells were positive for synaptophysin. The conclusive diagnosis was that of a pancreatic ganglioneuroma, specifically the mature subtype (Figure 3). The postoperative evolution went without any complication, the intra-abdominal drainage was removed early, and the patient was discharged six days postoperatively. During her last follow-up appointment in October 2023, the patient remained asymptomatic, and no tumor growth was confirmed by an abdominal CT scan.

## Discussion

Most retroperitoneal ganglioneuromas are asymptomatic tumors until they grow significantly and cause mass effects or obstructive symptoms, as observed in this case with abdominal distention and postprandial fullness. Familial predisposition is rarely observed but can be found in conditions such as Turner's syndrome, type 1 neurofibromatosis, and multiple endocrine neoplasia type IIb. Another way in which they can be symptomatic is by inducing a hypertensive crisis during surgery associated with the release of catecholaminergic peptides by secreting tumors [4]. Treatment options for these patients are limited, with surgical removal and adequate preoperative medication being the best option offered. The use of chemotherapy or radiotherapy does not present any proven benefits. Progression, late recurrence, or malignant transformation is rarely presented. For this reason, long-term periodic radiological follow-up is always required. The surgical procedure is necessary due to the presence of symptoms associated with a large-volume retroperitoneal mass. These tumors tend to involve and displace vascular structures but generally do not cause a narrowing of the lumen when there is involvement [5]. Encasement of the celiac trunk and superior mesenteric artery is mostly presented in advanced pancreatic tumors. Vascular encasement is defined as when the tumor surrounds the vessel by more than 180 degrees. This phenomenon has been utilized as a defined risk factor for the stratification of abdominal neuroblastoma by the International Neuroblastoma Staging System (INSS). However, there is ongoing discussion regarding the surgical approach and radicality in ganglioneuromas. It is important to mention that the first step to achieving this goal is accurate preoperative staging, typically conducted using contrasted intravenous (IV) high-resolution dynamic CT scans (with an accuracy of around 95%) [6-8]. 

**Figure 4:**
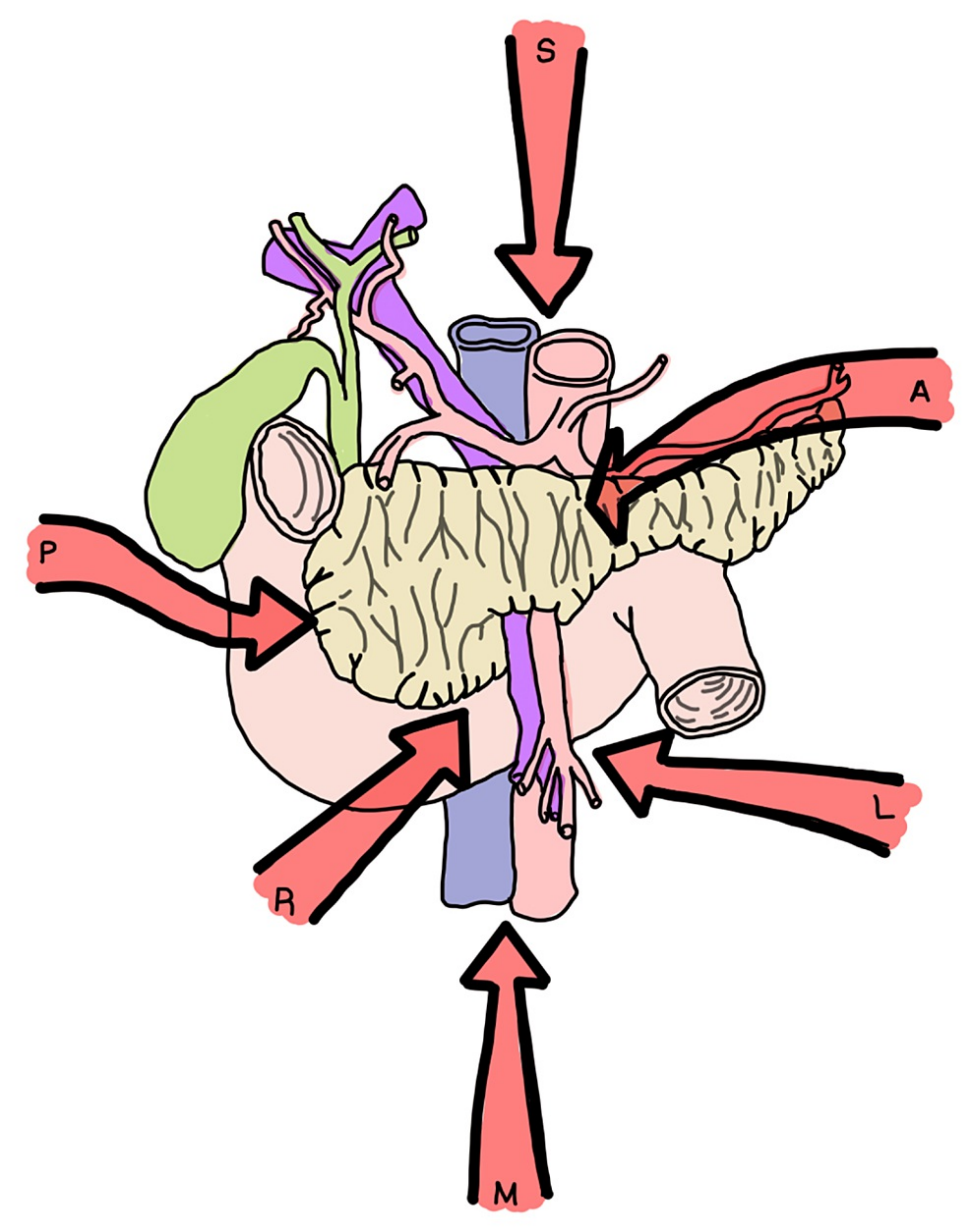
Artery-first surgical approach. Image credits: Natalia M. Barron Cervantes. Adapted from [3]. S, superior approach; A, anterior approach; P, posterior approach; L, left posterior approach; R, right or medial uncinate approach; M, mesenteric approach

Many surgical approaches have been used throughout medical history, but currently, the preferred method involves an open midline laparotomy, an extensive Kocher maneuver, and an artery-first approach. The goal is an R0 resection of the tumor with total vascular preservation to the greatest extent possible. In this surgical technique, the key point is to ligate all feeding arteries before the division of the pancreas, with the intent to reduce blood loss and be able to resect as much tumor as possible. During this procedure, the visualization of the ligament of Treitz needs to be performed first to access the retroperitoneal space and allow dissection of the superior mesenteric artery (Figure 4) [9]. To meet the goal of achieving negative margins, it is necessary to start with this artery and then proceed to the celiac trunk.

Although our objective will always be to obtain a negative histopathological margin, known as R0 resection, sometimes this is not achievable, and we need to perform an R1 or R2 resection. In R1 resection, there is no residual macroscopic tumor; however, histopathologic margins will be positive for the tumor. When referring to an R2 resection, there will still be macroscopically visible disease post-surgery [10]. In some cases, a trial dissection is necessary for patients in whom there is uncertainty about resectability. Particularly in these cases, the artery-first approach makes the most sense. An early evaluation of whether there is arterial involvement or not must be determined before committing to an irreversible step in the operation. An R2 resection rate has been reported at 20%, indicating that complete resection of the tumor is not always possible. However, this does not necessarily imply a worse prognosis [11]. In this scenario, partial resection is recommended to reduce the tumor volume and improve symptoms. However, long-term follow-up is necessary, because, despite benign behavior, there is a small tendency for malignant transformation. It is crucial to note that the allowance for this type of resection relies on the confirmation of a benign tumor through a histopathology report.

## Conclusions

Retroperitoneal ganglioneuroma is a rare diagnosis in general, which makes its suspicion and diagnosis difficult. In this case, its importance lies in its differential diagnosis and its complex diagnostic approach. Within the differential diagnoses, it is not only essential to rule out malignant pancreatic neoplasms, but it is also vital to evaluate the involvement of the pancreas and major vessels, such as the inferior vena cava, superior mesenteric artery, and celiac trunk. As mentioned earlier, R0 resection seems to be more of an anecdotal procedure in daily practice. This is demonstrated by multiple case reports where R2 resection is the most common, as in our case. The artery-first surgical approach has not only been the most widely used worldwide but also one of the approaches with the greatest theoretical support, which is why it was used in this case. One of the most important aspects to highlight in this case is that the use of complementary imaging studies, such as computed axial tomography, was key to determining the surgical approach for this patient. Likewise, it is of great importance to mention that the histopathological diagnosis that confirmed the benign nature of the tumor was what allowed an R2 resection to be performed and not expose the patient to the risk of vascular damage unnecessarily.
